# Methyltransferase-directed covalent coupling of fluorophores to DNA†
†Electronic supplementary information (ESI) available. See DOI: 10.1039/c6sc04229e



**DOI:** 10.1039/c6sc04229e

**Published:** 2017-03-14

**Authors:** Milena Helmer Lauer, Charlotte Vranken, Jochem Deen, Wout Frederickx, Willem Vanderlinden, Nathaniel Wand, Volker Leen, Marcelo H. Gehlen, Johan Hofkens, Robert K. Neely

**Affiliations:** a Department of Chemistry , KU Leuven , Celestijnenlaan , 3001 Heverlee , Belgium; b Institute of Chemistry of São Carlos , University of São Paulo , Brazil; c School of Chemistry , University of Birmingham , Edgbaston , Birmingham B15 2TT , UK . Email: r.k.neely@bham.ac.uk

## Abstract

We report an assay for determining the number of fluorophores conjugated to single plasmid DNA molecules and apply this to compare the efficiency of fluorophore coupling strategies for covalent DNA labelling. We compare a copper-catalyzed azide–alkyne cycloaddition reaction, amine to *N*-hydroxysuccinimidyl ester coupling reaction and strain-promoted azide–alkyne cycloaddition reaction for fluorescent DNA labelling. We found increased labelling efficiency going from the amine to *N*-hydroxysuccinimidyl ester coupling reaction to the copper-catalyzed azide–alkyne cycloaddition and found the highest degree of DNA labelling with the strain-promoted azide–alkyne cycloaddition reaction. We also examined the effect of labelling on the DNA structure using atomic force microscopy. We observe no distortions or damage to the DNA that was labeled using the amine to *N*-hydroxysuccinimidyl ester and strain-promoted azide–alkyne cycloaddition coupling reactions. This was in contrast to the copper-catalyzed azide–alkyne cycloaddition reaction, which, despite the use of copper-coordinating ligands in the labelling mixture, leads to some structural DNA damage (single-stranded DNA breaks).

## Abstract 2

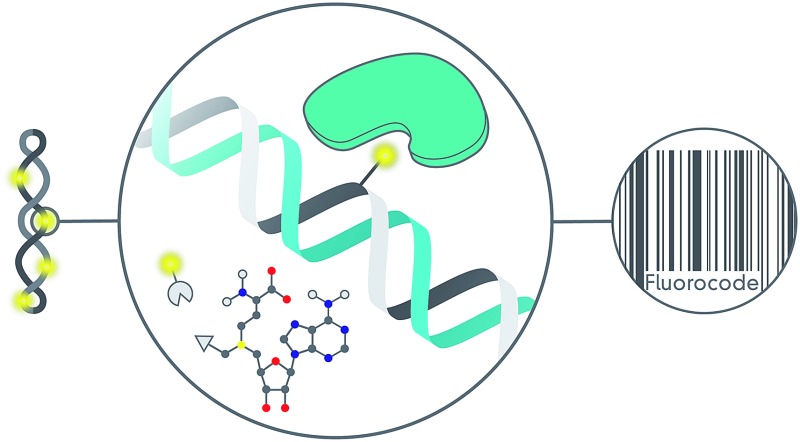
Highly efficient DNA labelling using an enzymatically-directed, strain-promoted azide–alkyne cycloaddition.

## Introduction

**Fig. 1:**
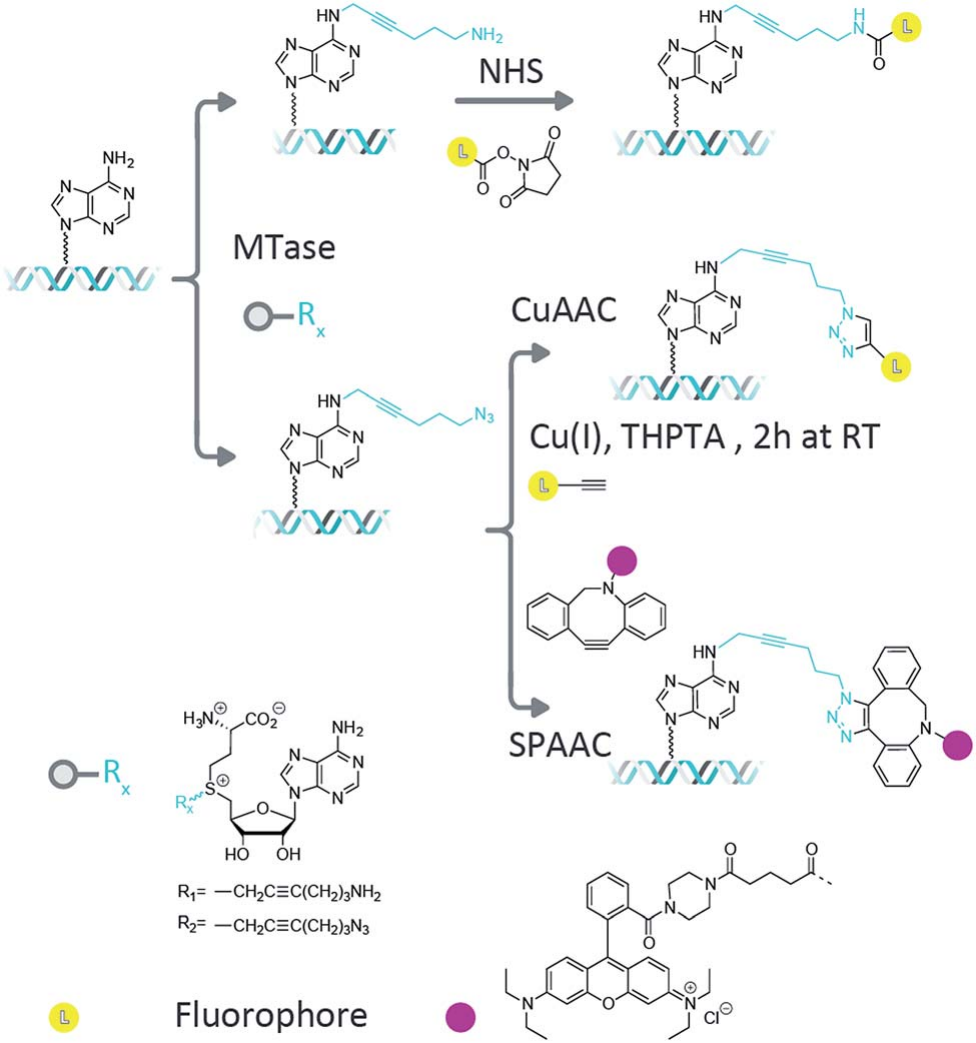
Two step labelling scheme. In the first step, the DNA is transalkylated using a DNA MTase and an AdoMet analogue which results in the DNA molecules carrying functional groups at known loci. Amine (R1) functionalized DNA can be coupled to a fluorophore using NHS ester chemistry, whereas for azide (R2) functionalized DNA this can be done using CuAAC and strain-promoted azide–alkyne cycloaddition (SPAAC) reactions.

Methyltransferase enzymes (MTases) are finding increasing applications in the targeting of fluorophores and other modifications to biological molecules.^1^ This is because, compared to untargeted chemical modifications, enzyme-directed labelling modifies the substrate of interest in an efficient and site-specific fashion. In nature, there are hundreds of known (and thousands of putative) methyltransferase enzymes whose function is to catalyze the transfer of the methyl group from their ubiquitous cofactor, *S*-adenosyl-l-methionine (AdoMet), to DNA, RNA and protein targets.^2,3^ In prokaryotes, the DNA methyltransferase enzymes are key components of the defence mechanism used by bacteria to protect themselves from invasion by foreign bacteriophage DNA.^4^ Their DNA methylation is therefore targeted and efficient, occurring at short sequences, typically between four and eight base pairs in length.^5^ Three different type of methylations are commonly found; cytosine C5, cytosine N4 (exocyclic amine) and adenine N6 methylation. The methyltransferase enzymes can catalyze DNA transalkylation reactions with extended and complex chemical moieties. These reactions are enabled by synthetically-prepared AdoMet analogues and can be broadly divided into two groups, aziridine-substituted adenosines^6^ and doubly-activated AdoMet analogues.^7^ The aziridine-based cofactors were described by Weinhold *et al.* ^8^ and allow direct, enzymatic coupling of a fluorophore to a DNA molecule.^9,10^ However, a disadvantage of this technique is the need for stoichiometric amounts of the methyltransferase enzyme in the reaction mixture. This problem has been overcome through the use of doubly-activated cofactors (methyltransferase-directed transfer of activated groups (mTAG)) where the methyl group of AdoMet is replaced by an extended chemical moiety.^7,11^ For these transalkylation reactions, the enzyme functions catalytically and is typically able to make several turnovers of the cofactor molecules per minute (though this rate is typically a factor of ten slower than the methylation reaction and can varying significantly depending on the enzyme–cofactor combination). The two-step mTAG approach is illustrated schematically in Fig. 1. Recent work by the Weinhold group has demonstrated the single-step transfer of a fluorophore with the M.TaqI methyltransferase enzyme to DNA, though no synthetic details on the preparation of this molecule were given.^12^ We previously used the mTAG labelling approach in single-molecule DNA mapping experiments. We found that whilst the methyltransferase-directed functionalization of the DNA was complete, the fluorophore-coupling step was relatively inefficient. Amine to *N*-hydroxysuccinimidyl ester (NHS) coupling reactions resulted in low DNA labelling efficiencies (30–40% at the single-molecule level)^13^ and the CuAAC reaction resulted in a labelling efficiency of 60–70%.^14^ Furthermore, these reported labelling efficiencies are for the single DNA molecules that were selected from the sample for DNA mapping analysis; they are not representative of the overall fluorophore coupling efficiencies across the entire DNA population.

Measuring fluorophore coupling efficiencies represents a significant technical hurdle, since the working DNA concentrations are relatively low (typically nanomolar for large, plasmid or genomic molecules). Hence, the simple quantification of dye coupling efficiencies using absorption spectroscopy is not possible. Here we set out to address this issue using a single-molecule counting approach that allows us to make quantitative comparisons of the labelling efficiency across a population of many thousands of large, plasmid DNA molecules with complex topologies.

We also take the opportunity to examine the effect of fluorophore coupling on DNA topology using an atomic force microscope.^15^ Notably, in previous studies we have found that DNA is damaged in the copper-catalyzed azide alkyne cycloaddition (CuAAC) reaction. We applied an AFM-based assay to investigate the influence of the coupling reaction on the DNA structure, again allowing single-molecule, quantitative assessment of the extent of the DNA damage within a population of molecules.

## Materials and methods

All materials were purchased from Sigma Aldrich and used as received unless stated otherwise.

### Synthesis of dibenzylcyclooctyne (DBCO) Rhodamine B

Rhodamine B piperazine amide was synthesized following the procedure described by Nguyen *et al.* ^16^ Rhodamine B was ring closed under basic conditions (NaOH) and added to a mixture of trimethyl aluminium and piperazine. DBCO acid (5-(11,12-didehydrodibenzo[*b*,*f*]azocin-5(6*H*)-yl)-5-oxopentanoic acid) was prepared in 7 steps starting from dibenzosuberenone as previously described by Sachin *et al.* ^17^ DBCO acid (0.15 mmol) was dissolved in 1 ml of dry dimethyl formamide in a flame dried flask and the solution was flushed with nitrogen for 5 min. Triethylamine (0.92 mmol) and HBTU (2-(1H-benzotriazol-1-yl)-1,1,3,3-tetramethyluronium hexafluorophosphate; 0.46 mmol) were added to the reaction mixture and the reaction mixture was stirred for 2 h at room temperature. Rhodamine B piperazine amide (0.23 mmol) was added and the reaction mixture was stirred for another 1.5 h. The reaction was followed with thin layer chromatography (9/1: dichloromethane/methanol). The reaction mixture was concentrated under reduced pressure and purified using column chromatography (95/5: dichloromethane/methanol) and DBCO Rhodamine B was obtained (7 mg, 5%); ^1^H NMR (Fig. S1,† 600 MHz, 353K, DMSO-*d*_6_): *δ* 7.76 (m, 2H), 7.67 (m, 1H), 7.62 (m, 1H), 7.51 (m, 2H), 7.44 (m, 2H), 7.33 (m, 1H), 7.29 (m, 1H), 7.26–7.13 (m, 4H), 7.09 (m, 2H), 6.93 (m, 2H), 5.07 (d, *J* = 14.04 Hz, 1H), 3.79 (m, 1H), 3.66 (q, *J* = 6.90 Hz, 8H), 3.20 (m, 8H), 2.17 (m, 1H), 1.93 (m, 2H), 1.82 (m, 1H), 1.48 (m, 2H) and 1.22 (m, 12H); ^13^C NMR (Fig. S2,† 600 MHz, 353K, DMSO-*d*_6_): *δ* 171.5, 170.1, 166.6, 157.1, 155.1, 151.8, 148.4, 135.2, 132.4, 131.8, 130.6, 130.4, 129.8, 129.7, 129.6, 129.4, 128.9, 128.0, 127.9, 127.6, 127.5, 126.7, 125.0, 122.4, 121.3, 114.3, 113.1, 108.3, 54.7, 48.6, 45.4, 38.2, 33.3, 29.0, 20.5 and 12.4; solvents peaks (*e.g.*, DMF, DMSO and water) and their satellites were observed in the NMR spectra, as well as some small impurities (*e.g.*, TMU). Despite these minor impurities, the compound is sufficiently pure to be used as a reagent for DNA labelling in copper-free click reactions. This has been demonstrated in a model reaction with Ado-6-azide labeled DNA, which was successfully coupled to the DBCO Rhodamine B compound. ESI HRMS calculated for C_52_H_54_N_5_O_4_ [M]^+^: 812.41700; found: 812.4160, Fig. S3.†

The Ado-6-amine and Ado-6-azide cofactor analogues were synthesized as reported by Lukinavičius *et al.* ^18^

### Preparation of sequence-specifically modified DNA

Plasmid DNA (pUC19, NEB) (50 ng μL^–1^), an AdoMet cofactor analogue (150 μM Ado-6-amine or Ado-6-azide), and M.TaqI DNA methyltransferase (0.1 mg ml^–1^) were incubated in CutSmart buffer (New England Biolabs) for 2 h at 60 °C. Subsequently, 1 μL of proteinase K was added and the reaction was incubated for 1 h at 55 °C. The product was purified using silica-based columns (DNA Clean and Concentrator-5, Zymo Research) and eluted with 25 μL of Milli-Q water.

### Fluorescent labelling using an amine-to-NHS ester coupling

In a 50 μL reaction, 0.89 μg of amine modified pUC19 DNA was mixed with 0.01 M phosphate buffered saline (Sigma Aldrich), 500 μM Atto-647N-NHS dye (Atto-Tec) and a volume of DMSO corresponding to 5, 10, 25 and 30% of the solution by volume. The reactions were incubated for 2 h at room temperature and purified using silica-based columns (DNA Clean and Concentrator-5, Zymo Research). The fluorescently labeled DNA was eluted using the elution buffer provided by the manufacturer.

### Fluorescent labelling using a CuAAC reaction

A solution with a volume of 100 μL containing 25% DMSO, 200 μM CuSO_4_, 2 mM tris(3-hydroxypropyltriazolylmethyl)amine (THPTA) (Sigma-Aldrich) and 200 μM Atto-647N-propargylamide (Atto-Tec) was prepared and to this was added 0.86 μg of the azide labeled DNA. To trigger the coupling reaction, 5 mM of a freshly prepared sodium ascorbate (Sigma-Aldrich) solution was added to the reaction mixture. The sample was incubated at room temperature for 30 minutes and subsequently purified using silica-based columns (DNA Clean and Concentrator-5, Zymo Research).

### Fluorescent labelling using an SPAAC reaction and the solvent effect

The SPAAC reaction was performed in a variety of different solvents and solvent mixtures (water, ethanol, DMF and DMSO (Sigma-Aldrich)). 1 μg of azide coupled pUC19 DNA was incubated with 50 μl of the cyclooctyne–rhodamine dye at a concentration of 1 mM. The reaction mixtures were incubated overnight at room temperature and then purified using a silica-based column (DNA Clean and Concentrator-5, Zymo Research) and eluted with water.

### The fluorescence microscopy setup

The images were acquired on an inverted microscope (Olympus IX83), using either 488 nm (YOYO-1), 561 nm (Rhodamine B) or 640 nm (Atto647N) fibre-coupled lasers and total internal reflection illumination. An Olympus quad-band dichroic mirror (405/488/561/640) was used to direct laser light to the sample and collect the fluorescence emission. We used an Olympus UAPON 150x TIRF objective and Hamamatsu, ImagEM EM-CCD camera for imaging.

### Sample preparation

Coverslips (0.13 mm, 22 × 22 mm, VWR International) were cleaned by rinsing with deionised water, dried with argon gas and stored overnight in a muffle furnace at 450 °C. The coverslips were coated with poly-l-lysine (PLL) (0.01% w/v in H_2_O) for 15 minutes to allow adsorption, rinsed with deionised water and carefully dried with argon gas. 50 μL of a DNA solution (fluorescently labelled DNA at a final concentration of ∼1 ng μL^–1^), in a buffer (pH 7.70) containing 50 mM Tris, 50 mM NaCl and 1 mM EDTA, was spin-coated onto the PLL-coated coverslip at a rotation speed of 2500 min^–1^. While spinning, the coverslip was rinsed with 5 mL of deionised water, added in a dropwise fashion.

### Imaging

A perfusion chamber (8–9 mm diameter × 0.9 mm depth, Grace Bio-Labs) was sealed onto the DNA-coated coverslip. 45 μL of phosphate buffered saline (Sigma Aldrich) solution containing 5 nM of the intercalating dye YOYO-1 (Life Technologies) and 50 mM of β-mercaptoethylamine (Sigma-Aldrich) was introduced to the chamber immediately prior to imaging. The imaging was performed according to a previously reported binding-activated localization microscopy (BALM) approach.^19^

For each system, at least five movies were acquired from different regions of the same sample. First, between 250 and 1000 frames were recorded in a so-called bleaching experiment using 640 nm excitation light for the Atto-647N dyes and 561 nm excitation light for the rhodamine B dyes. Movies were recorded until all fluorophores in the field of view had been photobleached. Following this, a 488 nm laser was used to first photobleach the bound YOYO-1 dyes for 10 s, then acquire 2000 frames of reversible YOYO-1 blinking/binding. All imaging sequences used an exposure time of 30–50 ms.

### Data analysis

The data was analyzed using a previously-developed ‘bleaching analysis’ approach.^13,20^ Conceptually, we start the analysis from the last frame of a movie in which all of the emitters are bleached and play the movie in reverse. Emitters that ‘appear’ in the reversed movie are identified and their images are fitted with a 2D Gaussian profile, which is then used to subtract the image of the emitter from the remaining frames of the movie. In this fashion, we are able to count the number of bleaching events in the movie.

In order to associate bleaching events with plasmid DNA molecules, movies of the YOYO-1 dye reversibly binding to deposited DNA plasmids were recorded and analyzed using localization analysis. The identified emitters were convolved with a point-spread function determined using the localization error to produce a super-resolution image of the plasmid structure. All images were analyzed using the Localizer^21^ plugin for IgorPro.

Finally, to count the number of labels that each plasmid is carrying we make a binary image of the plasmids using the super-resolution (YOYO-1 derived) data, which is used to describe the size and shape of a given molecule. Then, we define intact plasmids as those that cover an area greater than 0.15 μm^2^ with a circularity less than 1.7, where the circularity is described by
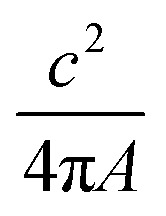
where *c* is the feature’s perimeter and *A* is its area. For each of the plasmids that pass the circularity test, coincident localized emitters from the bleaching experiment (MTase-catalysed labelling) are identified and counted. Finally, we prepare a histogram of the results to show the number of plasmids in a population with ‘*n*’ attached fluorophores. The Matlab code for fluorophore counting is available on request.

### AFM measurements

Plasmid DNA samples were diluted to a final concentration of ∼0.5 ng L^–1^ in a buffer containing 200 mM Na-acetate and 10 mM Tris–HCl (pH = 8.0). A 20 μL solution was deposited onto poly-l-lysine (0.01% w/v)-coated mica for 30 seconds before the sample was gently rinsed with deionised water (20 mL) and dried using a gentle flow of argon gas.

The dried samples were measured in air using amplitude-modulation atomic force microscopy with a commercial multimode AFM equipped with a nanoscope VIII controller and a J-scanner (Bruker). Silicon cantilevers (AC160TS; Olympus) were excited at ∼300 kHz and the feedback parameters were adjusted to apply minimal tip-sample interaction forces and to allow stable imaging. The image processing was performed using a Scanning Probe Image Processor (v6.3.; Image Metrology) and involved background subtraction including 3rd degree polynomial global correction, and line-by-line correction using the histogram alignment routine. The images were acquired with a field of view of 2–4 μm^2^ (1024 × 1024 pixels). Around 150 molecules were recorded and their morphology was classified based on the number of nodes, which we counted manually.

For control experiments, 1 μg of pUC19 plasmid DNA was incubated in NEB3.1 buffer with 1 unit of Nt.BspQI (New England Biolabs) for 1 hour at 50 °C. The enzyme was then inactivated by incubating it for 5 minutes at 65 °C and the DNA was purified using a Genejet PCR purification kit (Thermo Fisher Scientific).

## Results and discussion

We collected several large datasets that would allow us to count the number of fluorophores on thousands of individual plasmid DNA molecules in a straightforward manner. We employed the pUC19 plasmid in these experiments, which contains 4 recognition sites for the M.TaqI (5′-TCGA-3′) MTase enzyme. Since these sites are palindromic, each site can potentially carry two functional groups and hence, in principle, up to 8 fluorophores can be attached to an individual plasmid molecule.

After methyltransferase-directed DNA modification with the Ado-6-amine and Ado-6-azide cofactors and M.TaqI the plasmids showed complete protection from restriction digestion by the R.TaqI (restriction) enzyme (ESI Fig. S4 and S5†), indicating that for each transalkylation reaction there is at least one modification of the DNA per recognition site (only hemi-methylation of the 5′-TCGA-3′ sites is required to prevent DNA digestion by the R.TaqI enzyme). Fluorophores are subsequently conjugated to these sites using either amine-NHS,^22^ CuAAC^23^ or SPAAC^24^ coupling chemistry, as shown schematically in Fig. 1. These methods are generally regarded as straightforward reactions that can be readily performed under aqueous conditions. The amine–NHS coupling targets (non-aromatic) primary amine groups for peptide bond formation and results in the formation of a stable peptide bond. A primary concern for this reaction is the (necessary) instability of the NHS ester towards hydrolysis, which competes with the peptide bond formation.^25^ The copper-catalysed azide–alkyne cycloaddition is an efficient, bio-orthogonal reaction that has been used extensively for modifying DNA.^26^ However, we have found that whilst any DNA damage by Cu(i) can be limited with the addition of a coordinating ligand (tris(3-hydroxypropyltriazolylmethyl)amine, THPTA) to the reaction, damage is not completely prevented and this becomes particularly problematic for large DNA molecules.^14^ The strain-promoted azide–alkyne cycloaddition, by contrast, is a metal free alternative to the copper-catalysed cycloaddition that we selected because of its simple and specific application in bio-conjugation reactions. Note that despite the different reaction conditions we employ, the fluorescent dye is always in (>1000-fold) excess in the reaction.

Following the fluorescent labelling, the DNA is purified and deposited on a surface and imaged in two colors to characterize its shape and the number of methyltransferase-directed labels that it carries, using fluorescence microscopy. In practice, this is achieved firstly through using the DNA intercalating dye YOYO-1, which binds non-specifically to the DNA, as a way to characterize plasmid size/shape and secondly by counting (using photobleaching) the number of fluorophores attached to the MTase-functionalized sites on each of the identified plasmid molecules.

**Fig. 2:**
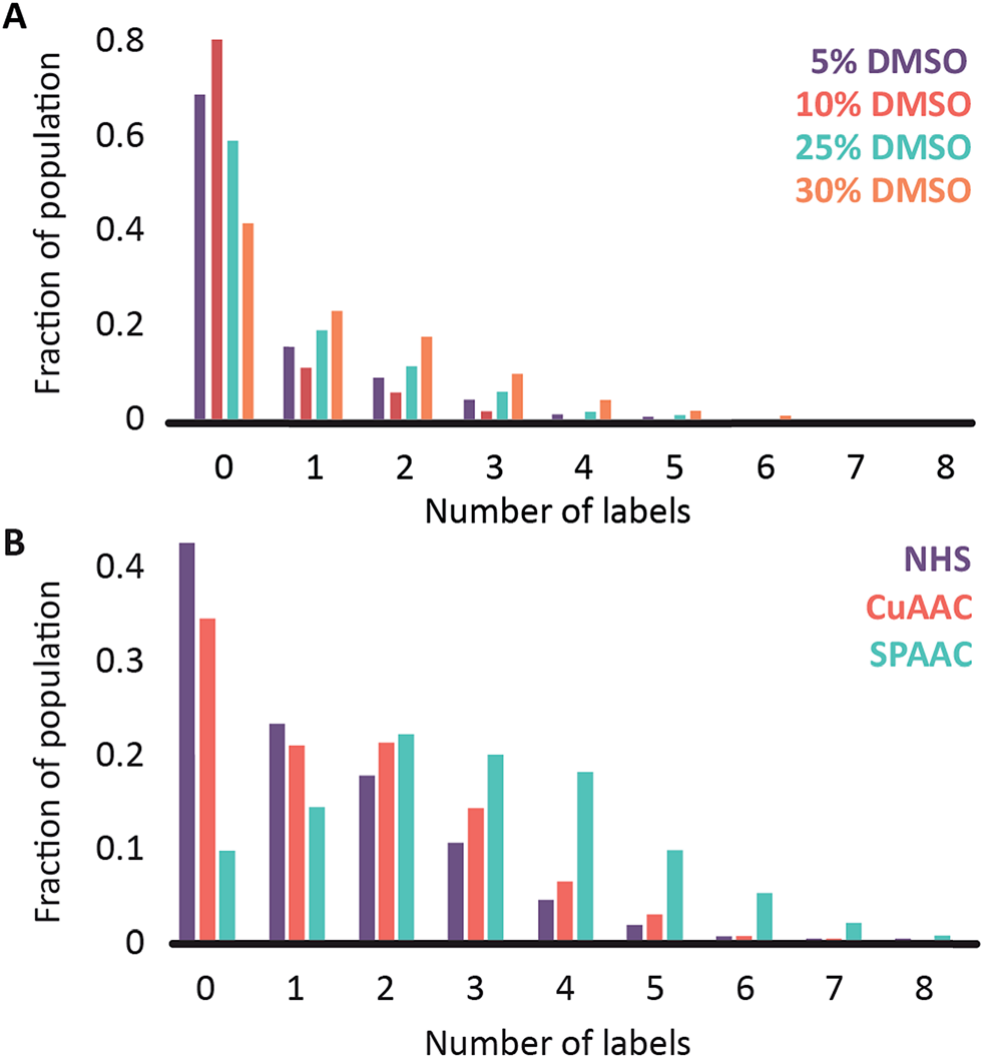
Histograms showing (A) the labelling efficiency for the NHS ester-to-amine coupling reaction as a function of the DMSO concentration in the coupling reaction and (B) a comparison of three different coupling chemistries: NHS ester-to-amine (in 30% DMSO, purple), CuAAC (in 25% DMSO; red) and SPAAC (in 25% DMSO; turquoise).

**Fig. 3:**
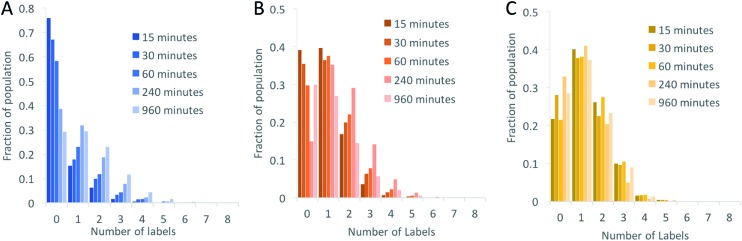
Histograms showing the labelling efficiency as a function of reaction time for (A) the NHS ester to amine coupling reaction, (B) the copper-catalysed cycloaddition reaction (CuAAC) and (C) the strain-promoted cycloaddition reaction (SPAAC). Note the *y*-axes are scaled differently.

We began by investigating the DNA labelling efficiency achieved using the NHS–amine coupling reaction. Fig. 2A shows that the fluorophore (Atto647N) coupling using this approach is remarkably inefficient. The coupling efficiency can be marginally improved with the addition of DMSO to the dye-coupling reaction, which likely improves the solubility of the dye. Even in the best case (30% DMSO in the coupling reaction), we found that 40% of the plasmid molecules carried no fluorophore following this treatment and the population as a whole carried an average of only 1.2 labels per plasmid (a two hour coupling reaction). The ineffective nature of this coupling reaction is surprising, though a recent study using mass spectrometry to investigate the coupling efficiency of a fluorescent dye (Atto655) to a peptide found the reaction to be similarly inefficient, with a reported degree of labelling of 40%.^27^ We also followed this reaction over time, observing that the fluorophore coupling reaction reaches completion on a timescale of several hours (Fig. 3A). Under the pseudo first order conditions we apply, the rate of the fluorophore coupling reaction is calculated as 1 × 10^–4^ s^–1^, with the second order coupling rate being 0.2 M^–1^ s^–1^. The coupling rate is calculated here based on the average number of fluorophores per plasmid at each time point, Fig. S8.† With the reaction allowed to continue for 16 h, we see an average of 1.4 labels per plasmid molecule.

In earlier DNA mapping experiments, we observed an improved fluorophore coupling efficiency when using the CuAAC reaction, as compared to the NHS–amine coupling.^13,14^ Fig. 2B shows that, indeed, we see a slight improvement in the overall labelling efficiency in the CuAAC reaction, to an average of 1.5 labels per plasmid. This is not as great an improvement as might have been expected from our DNA mapping measurements and we attribute this to the fact that in the present study we consider the entire ensemble of DNA molecules in the sample and not just a subset of well-labeled molecules. The kinetics analysis of this reaction was challenging to follow for reaction times longer than 1 hour (Fig. 3B). This is a result of the fluorophore counting being sensitive to the plasmid shape/topology. As we will show, the CuAAC reaction leads to significant DNA damage over time, which complicates our kinetics analysis when we allow the reaction to proceed for more than one hour.

A significant improvement in the fluorophore coupling efficiency is observed for the strain-promoted azide–alkyne cycloaddition (SPAAC) reaction. Here, less than 10% of the plasmid molecules are unlabeled and the plasmids in this sample carry a mean of 2.9 fluorophores. The kinetics analysis of this reaction shows that it was complete before the first time-point (15 minutes) we collected (Fig. 3C).

This data reveals two surprising results. The first is that the average labelling efficiencies appear low, relative to previous observations from DNA mapping experiments and reported coupling efficiencies for each of the reactions we applied. The second is that we see a broad range of labelling efficiencies at the single molecule level, with some plasmids carrying no labels whatsoever, whereas others have many.

The absolute numbers of fluorophores per plasmid are relatively low for all the coupling reactions. This, combined with the discrepancy between the present data and previous DNA mapping data, suggests that most of the palindromic target sites for M.TaqI carry only a single modification. In our DNA mapping experiments we recorded only that a site had been labelled, not the number of labels at a given site. High labelling efficiencies in mapping indicate that the majority of sites carry at least one label. Here, however, we reveal a more complete picture of the labelling system and observe much lower average labelling efficiencies. Hence, we hypothesize that the enzymatic modification of both of the adenine bases at the palindromic recognition site for the M.TaqI enzyme is rare. Potential causes for this would be, for example, a low binding affinity of M.TaqI for a site carrying a single modification.

The broad distribution of fluorophore counts that we see in a population of DNA molecules can be attributed to two factors: the efficiency of the enzymatic reaction and the fluorophore coupling efficiency. Since we have verified that the enzyme transfers at least four functional groups to DNA using the R.TaqI restriction enzyme, labelling efficiencies worse than this must be due to poor fluorophore coupling efficiencies.

The fluorophore coupling efficiency can be sub-optimal as a result of either extremely slow reaction kinetics (the reaction does not reach completion) or a competing process (such as methylation or fluorophore photobleaching) that leads to the active prevention of fluorescent labelling. We can model this behavior using a simple kinetics scheme: (eqn (1)) a fluorophore (F) is coupled, with a rate of *k*_b_, to one of *j* possible target sites on a plasmid (P) or (eqn (2)) a competing process occurs that renders a fluorophore invisible or unreactive towards a target site on the plasmid at a rate of *k*_d_:1
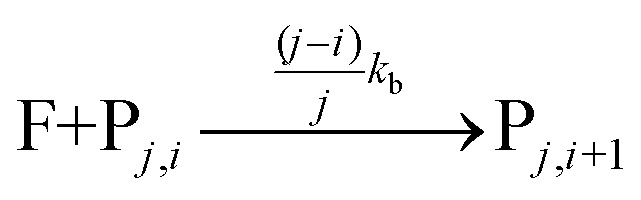

2
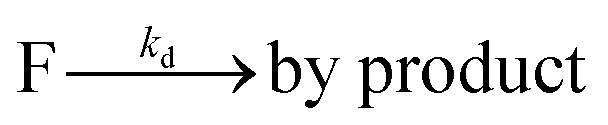



Modelling with pseudo-first order reaction conditions (a 20-fold excess of the reactive dye with respect to the target sites on the plasmids) gives labelling distributions, similar to those we observe experimentally, that critically depend on the rate and relative duration of the reaction and the ratio of the coupling and deactivation rate constants (*k*_b_[P]/*k*_d_) (Fig. S9†).

In the most-simple experimental case, the SPAAC reaction, the reaction goes to completion extremely rapidly. In this case, we are only able to model the observed distribution of labelling efficiencies by inferring that the competing reaction has a rate, *k*_d_, that is faster or comparable to that for the coupling, *k*_b_. We attribute the limited coupling efficiency of the DBCO dye to the presence of native *S*-adenosyl-l-methionine (co-purified with the M.TaqI enzyme), which is rapidly employed by the enzyme to methylate, rather than alkylate, the DNA. Indeed, we see some protection of M.TaqI-treated DNA against digestion by R.TaqI in the absence of an added cofactor (Fig. S4†), which is consistent with this hypothesis.

We also note that, for the SPAAC reaction, by increasing the negative charge on the fluorophore (Fig. S10†) we significantly reduce its coupling efficiency. We infer that the coupling rate slows dramatically with this increase in negative charge and that the reaction does not approach completion on a timescale of ∼16 h.

As we have suggested, the ‘competing process’ with rate *k*_d_ can be attributed to one or more underlying physical causes. These are broadly covered by three possible processes: reactive group decomposition, such as hydrolysis of the NHS ester moiety meaning the dyes cannot couple to DNA; dye decomposition such as photobleaching or oxidation that renders the fluorophore invisible in our counter assay; target site blocking through methylation of the target sites by residual AdoMet in the methyltransferase solution.

Future work will focus on the engineering of the transferable moiety of our AdoMet analogues for improved coupling efficiencies, but presently, coupling efficiencies of 70% or more are attainable using uncharged or positively charged rhodamine derivatives, such as tetramethylrhodamine (TAMRA) and rhodamine B.

**Fig. 4:**
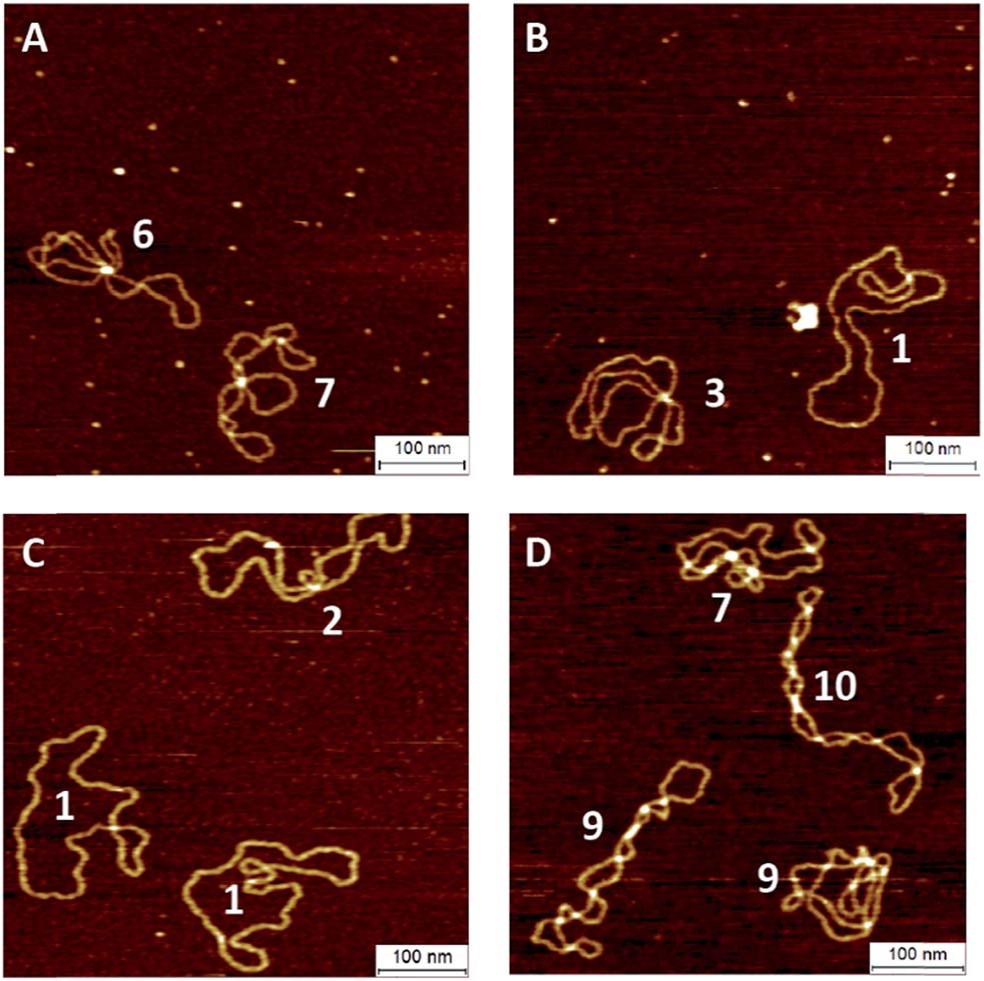
AFM images of pUC19 molecules in their supercoiled (A) and open-circular (B) forms and after the CuAAC (C) and SPAAC (D) reactions. The number adjacent to each molecule shows the determined number of nodes.

To investigate the influence of the coupling reaction on DNA integrity, we used AFM to determine the geometry of surface-adsorbed plasmid DNA (Fig. 4). Intact (covalently closed circular) plasmid DNA exists in a supercoiled state, wherein the double helix axis winds around itself as a result of torsional strain. When a single stranded break is generated in the supercoiled DNA, torsional strain is released, resulting in an open circular geometry. A double strand break linearizes the supercoiled circular DNA. These topological forms (supercoiled (SC), open-circular (OC) and linear (L)) can easily be distinguished in the AFM images. In particular, the open-circular and supercoiled topologies exhibit well-resolved distributions of intramolecular dsDNA crossings or nodes. The pUC19 molecules in their natural SC form exhibit a main peak centered at ∼7 nodes. This is in contrast to the OC form where the mean node number is decreased to ∼2, Fig. S11.† ^15^ The node number distributions of ensembles of plasmids thus reflect the extent of the DNA damage following a certain chemical treatment. We have used this approach to examine whether the attachment of a fluorophore introduces damage to the backbone of the DNA.

**Fig. 5:**
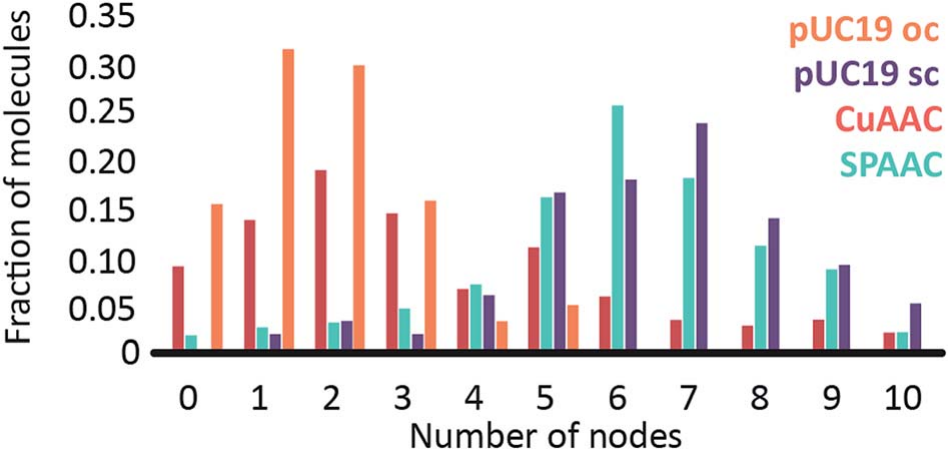
Graph showing the structural influence (by the distribution of node number) of the CuAAC (red) and SPAAC (turquoise) reactions on pUC19 molecules. A control reaction with untreated pUC19 molecules is represented by the purple bars, with a nicked, open circular control in orange.

The number of nodes observed for the plasmids that have been subject to the CuAAC and SPAAC reactions is compared in Fig. 5 to those counted for control experiments looking at SC and OC plasmid molecules. As expected, the majority of the plasmids are in the OC conformation following the CuAAC reaction, despite the presence of the THPTA copper-coordinating ligand and DMSO in the reaction. Conversely, the nodal distribution for the SPAAC ensemble closely matches that derived from the undamaged pUC19 control sample. Hence, we conclude that, in contrast to the CuAAC coupling reaction, DNA integrity is maintained throughout the SPAAC reaction. Furthermore, we see no obvious signs of distortion (*e.g.* kinking or crosslinking) of the DNA molecules as a result of the fluorophore coupling reactions.

## Conclusions

We have developed and applied a single-molecule, fluorescence assay to compare the relative efficiencies of DNA/fluorophore coupling reactions. We have shown that the NHS ester to amine and copper-catalyzed azide–alkyne cycloaddition reactions are rather inefficient compared to the strain-promoted azide–alkyne cycloaddition reaction. Whilst the SPAAC reaction proved to be the most efficient of the coupling methods, we found that in the ensemble of plasmids a broad distribution of labelling efficiencies exists. This we rationalize in terms of the reduced binding affinity of M.TaqI for an alkylated site (either because of the bulky first modification or slow dissociation of methyltransferase from its target site). Hence, although each site contains two target bases, only one is typically modified as lingering methyltransferase blocks the modification of the second target. The broad distribution of labelling efficiencies in a sample can be explained using a simple kinetics scheme involving competing DNA labelling and dye deactivation reactions. The inherent stability of the SPAAC reactants and the mild reaction conditions allow long reaction times and a relatively high degree of labelling with an average of 2.9 fluorophores per plasmid (>70% coupling efficiency, based on four sites per plasmid) without inducing DNA damage or other topological abnormalities. This represents an important step towards improving DNA labelling efficiencies and broadening the application of methyltransferase-directed labelling for DNA mapping and other biophysical experiments in the future.
