# Supplementary material for: Methyltransferase-directed covalent coupling of fluorophores to DNA
Source: Chem Sci. 2017 Mar 14;8(5):3804–11. doi: 10.1039/c6sc04229e (PMC5436541; doi:10.1039/c6sc04229e)
Supplement: Supplementary file 1 [file SC-008-C6SC04229E-s001.pdf]

Electronic Supplementary Material (ESI) for Chemical Science.
This journal is © The Royal Society of Chemistry 2017

Methyltransferase-directed covalent coupling of fluorophores to DNA

Milena Helmer Lauer, Charlotte Vranken, Jochem Deen, Nathaniel Wand, Wout Frederickx, Willem Vanderlinden, Volker Leen, Marcelo Henrique Gehlen, Johan Hofkens and Robert K. Neely

Supplementary Information

Materials and methods

Reagents and solvents were purchased from Sigma-Aldrich, and Acros and were used without further purification unless otherwise specified. M.*Taq*I (10 units/µl), *Taq*I (20 units/µl), pUC19 (1 µg/µl), SAM (32 mM solution in 5 mM H_2SO_4 and 10% EtOH), Proteinase K (800 units/ml), CutSmart buffer (pH 7.9) and loading dye were purchased from New England BioLabs.

Mass-Spectra were acquire on a quadrupole orthogonal acceleration time-of-flight mass spectrometer (Synapt G2 HDMS, Waters, Milford, MA). Samples were infused at 3uL/min and spectra were obtained in positive (or: negative) ionization mode with a resolution of 15000 (FWHM) using leucine enkephalin as lock mass.

General Enzymatic methylation-restriction experiment

1 µg pUC19 plasmid DNA was treated with 200 µM of cofactor and 1µl M.TaqI at 65°C for 0 min, 30 min, 1h and 2h in Cutsmart buffer in a total reaction volume of 20 µl. Subsequently, 1 µl of TaqI was added and the reaction mixture was incubated for another hour at 65°C. 1 µl of Proteinase K was added to the reaction and the reaction was incubated for 1h at 55°C. A positive control using SAM was performed under the same conditions. Negative controls in the absence of cofactor, in the absence of MTase and in the absence of both components were performed under similar conditions. The reaction volumes of all the controls was 40 µl of which 20 µl was treated with the restriction enzyme. A loading dye was added to all the samples and the reaction was analyzed using a 1% agarose gel and ethidium bromide staining.

Supplementary figures

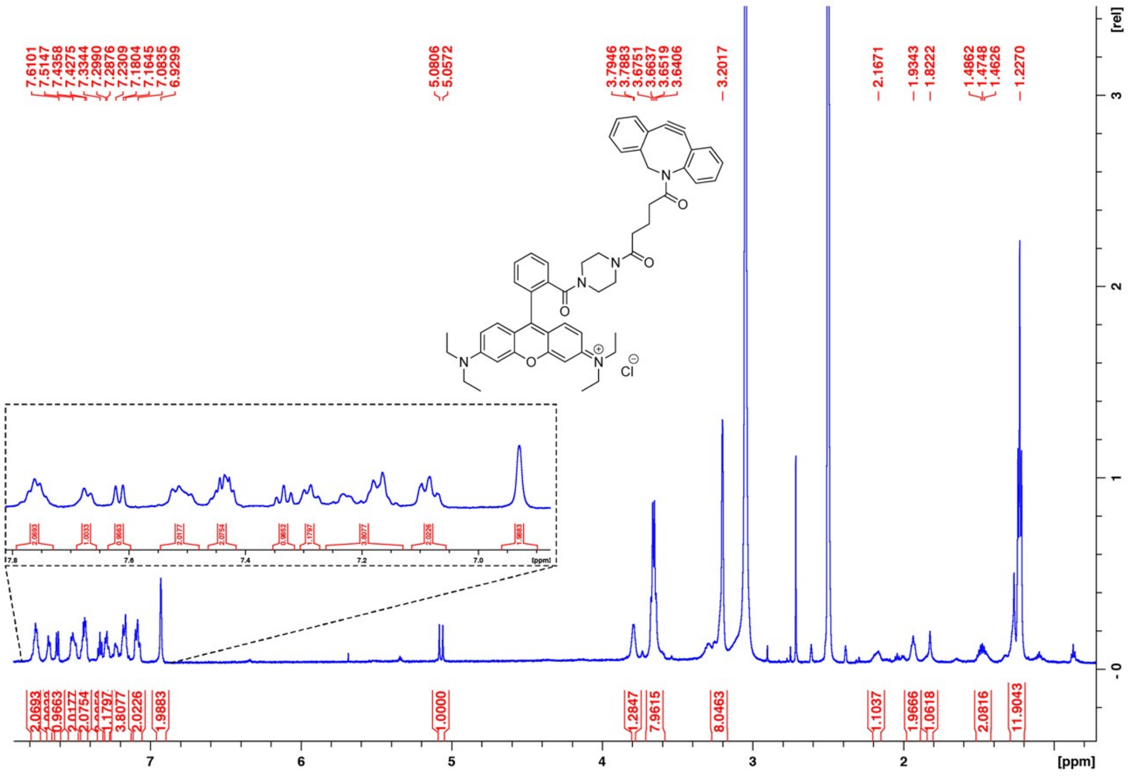

Figure S1: ^{1}H-NMR of DBCO Rhodamine B in DMSO-d_6.

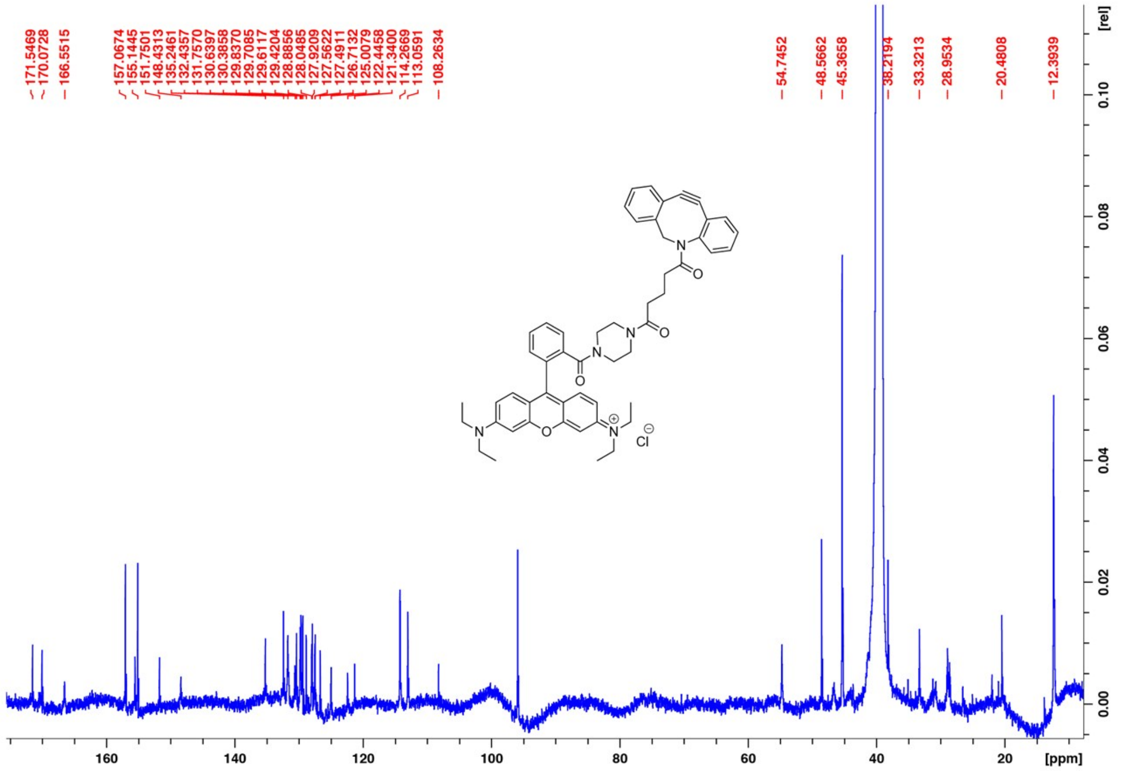

Figure S2: ^{13}C-NMR of DBCO Rhodamine B in DMSO-d_6.

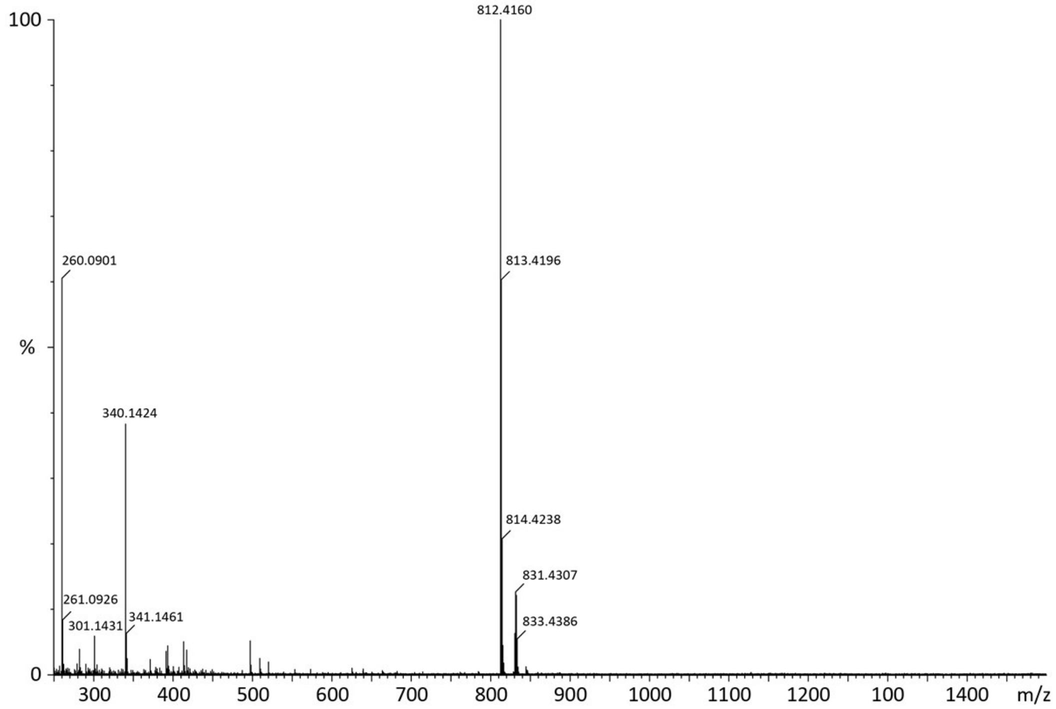

Figure S3: HR-ESI spectrum (+ve) of DBCO Rhodamine B. Calculated for $C_{52}H_{54}N_5O_4$ [M]+: 812.41700; found: 812.4160

DNA Protection assay by gel electrophoresis

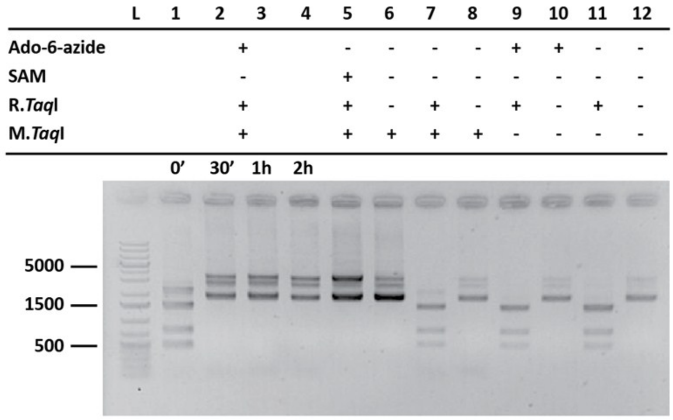

Figure S4: Methylation-restriction assay of Ado-6-azide cofactor, M.TaqI and pUC19

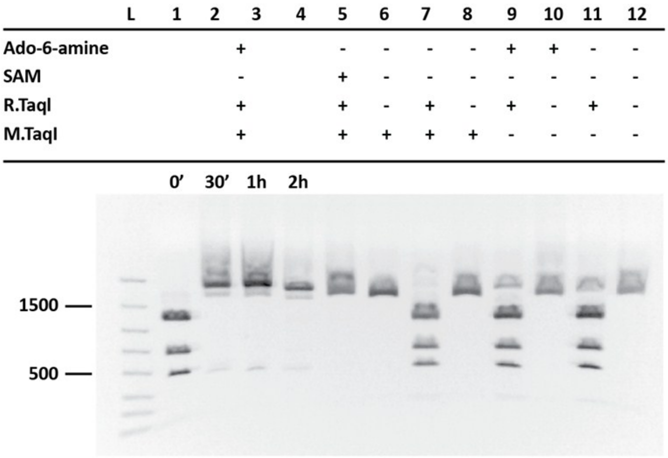

Figure S5: Methylation-restriction assay of Ado-6-amine cofactor, M.TaqI and pUC19

SPAAC – Effect of Solvents

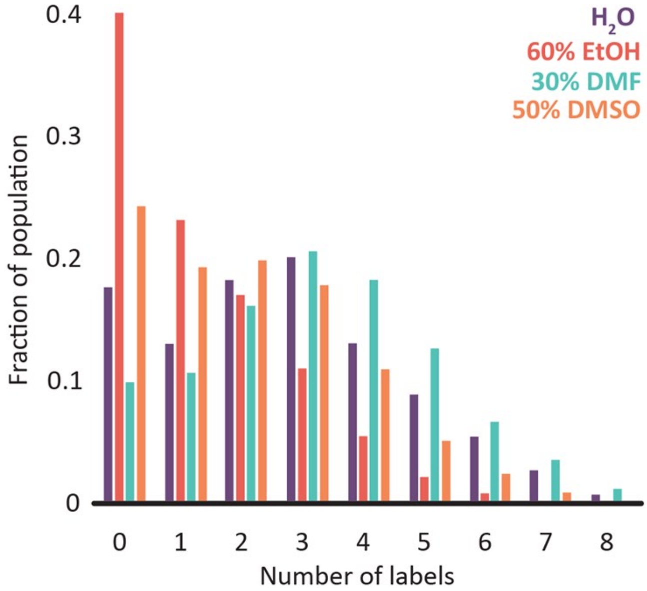

Figure S6: Effect of different solvents in the SPAAC reaction. H_2O (purple), 60% EtOH (red), 30% DMF (turquoise) and 50% DMSO (orange).

SPAAC – Effect of DMSO concentration

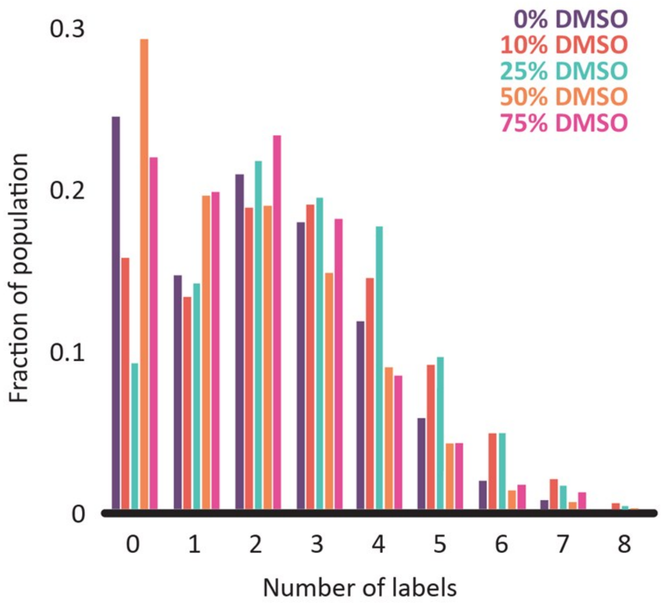

Figure S7: Influence of DMSO concentration on the SPAAC coupling efficiency. 0% (purple), 10% (red), 25% (turquoise), 50% (orange) and 75% (pink) DMSO concentrations were tested.

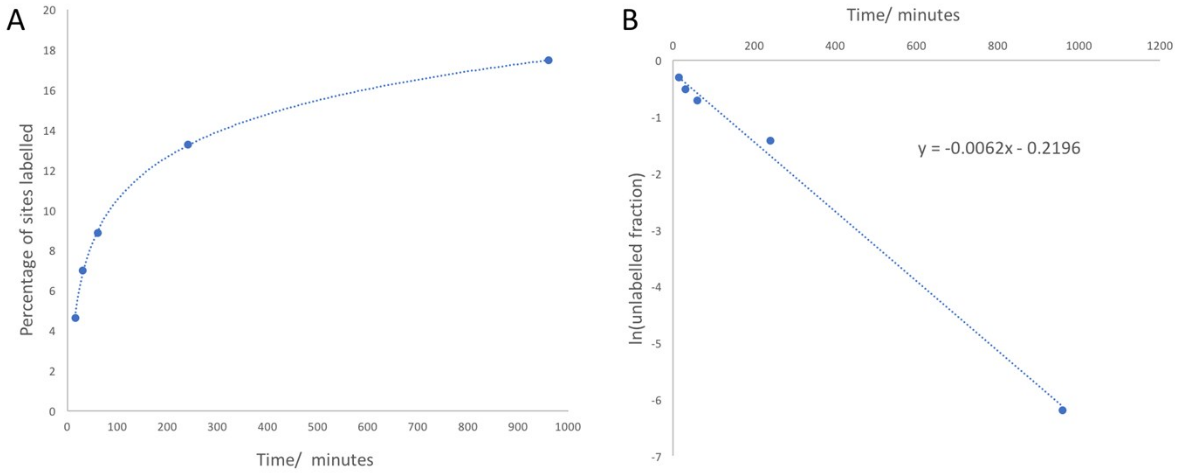

Figure S8: Reaction kinetics for the NHS-ester to amine coupling reaction followed using the single-fluorophore counting method. (A) Reaction progression, as a function of the overall labelling efficiency with time (B) Plot of the natural log of the decrease in the unlabeled fraction (assuming the reaction is complete at 17.5% labelling, from A) with time. Fitting a linear regression allows determination of the pseudo first order rate of the reaction.

Labeling efficiency simulation

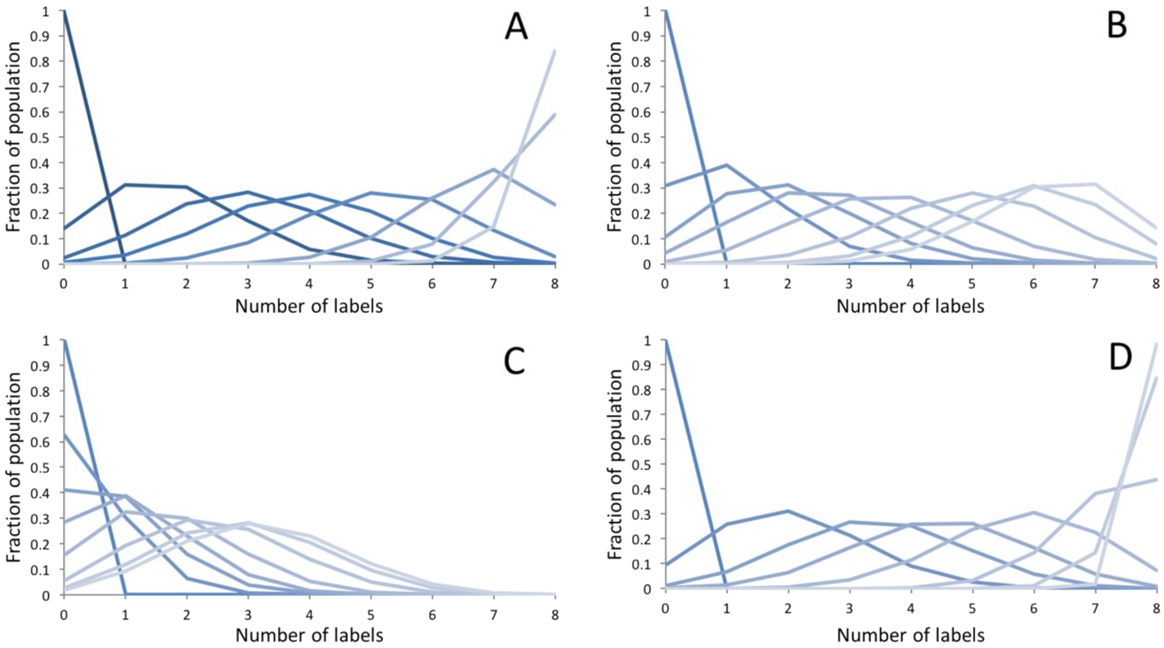

Figure S9: Simulation of dye to plasmid coupling efficiencies. In each case, the reactive fluorophore is in 20-fold excess and the maximum number of labels on a plasmid is 8. For each condition, the reaction progression is followed by the change in distribution of labelled plasmids from the dark-blue plot (left) to the lightest blue plot (right). All plots depict fluorophore distributions as determined by the model described by Equations 1 and 2 in the main manuscript. A: The coupling rate is five-times faster than the deactivation rate ($k_b = 5k_d$). B: The coupling rate is the same as the deactivation rate ($k_b = k_d$). C: The coupling rate is four times slower than the deactivation rate ($4k_b = k_d$). D: The coupling rate is slow and the rate of deactivation is zero. Resolution of the corresponding ordinary differential equation system was performed by fourth-order Runge-Kutta method.

SPAAC – Dye coupling efficiencies

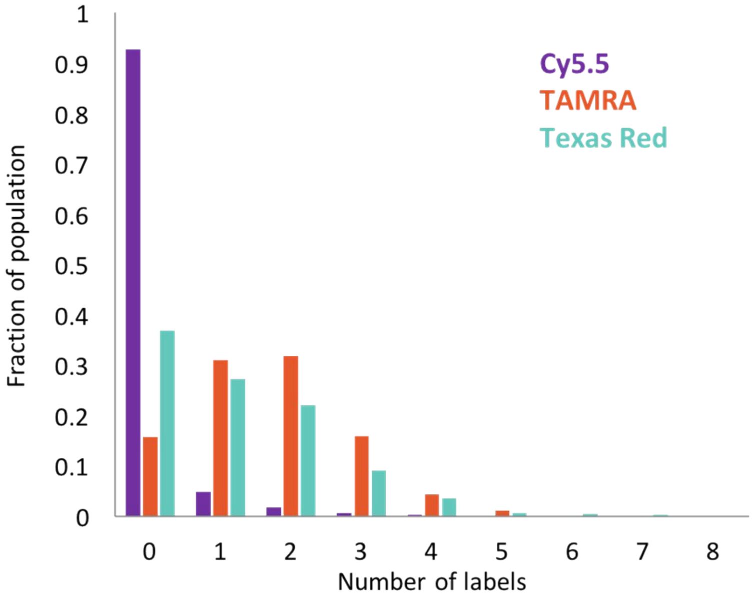

Figure S10: SPAAC coupling efficiencies for different commercially available DBCO-dyes. Cy5.5 (purple), tetramethylrhodamine (TAMRA) (orange) and texas red (turquoise) were tested. Coupling efficiencies negatively correlate with the extent of dye sulfonation.

AFM control experiments

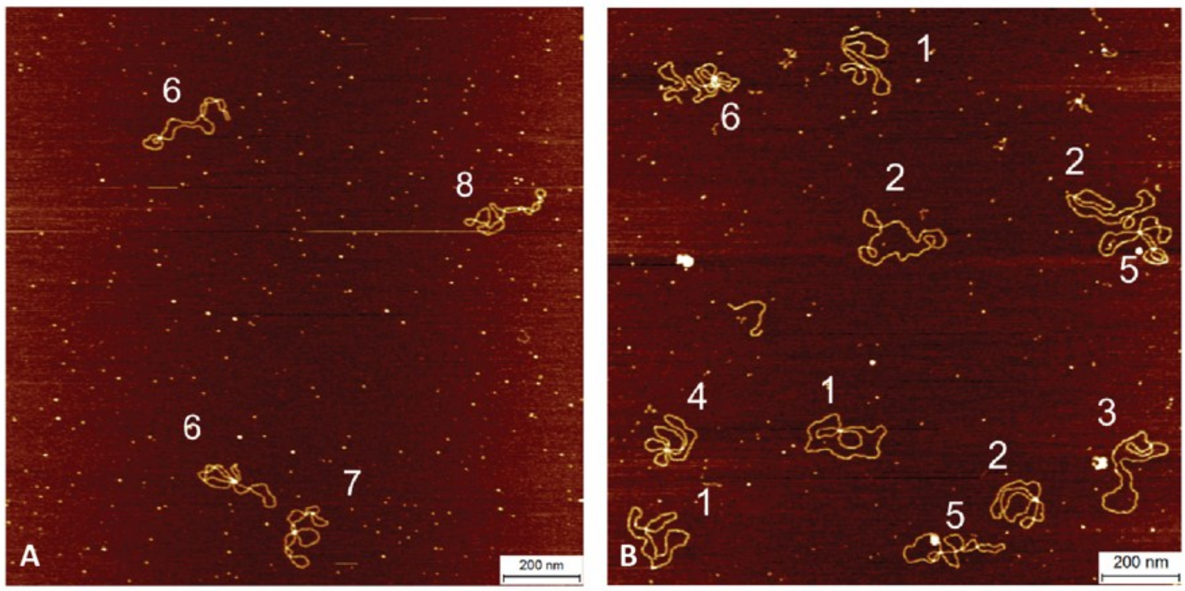

Figure S11: AFM control experiments. Left: pUC19 control in its natural supercoiled (SC) conformation; Right: pUC19 control in the open circular (OC) conformation.

AFM data

Table. Number of plasmids classified according to the number of nodes, after the fluorophore coupling by CuAAC or SPAAC reactions.

Number of nodes per molecule	pUC19 SC Control	pUC19 OC Control	CuAAC	SPAAC
0	0	10	27	3
1	4	20	42	5
2	7	19	58	6
3	4	10	44	9
4	13	2	20	14
5	37	3	33	32
6	40	0	17	51
7	53	0	10	36
8	31	0	8	22
9	20	0	10	17
10	11	0	6	4
Linear	0	0	32	0
**Total**	**224**	**64**	**308**	**200**

Classification of the plasmids morphology: where, open circular = from 0 to 4 nodes; supercoiled = more than 5 nodes.

	pUC19 control	CuAAC Standard	SPAAC
**Open circular**	28	191	37
**Supercoiled**	196	85	163
**Linear**	0	32	0
**Total**	224	308	200

	pUC19 control	CuAAC Standard	SPAAC
**Open circular**	12%	62%	18%
**Supercoiled**	88%	28%	82%
**Linear**	0	10%	0
